# Oxidative Stress Induced by Zearalenone in Porcine Granulosa Cells and Its Rescue by Curcumin *In Vitro*

**DOI:** 10.1371/journal.pone.0127551

**Published:** 2015-06-01

**Authors:** Xunsi Qin, Mingjun Cao, Fangnong Lai, Fan Yang, Wei Ge, Xifeng Zhang, Shunfeng Cheng, Xiaofeng Sun, Guoqing Qin, Wei Shen, Lan Li

**Affiliations:** 1 Key Laboratory of Animal Reproduction and Germplasm Enhancement in Universities of Shandong, Qingdao Agricultural University, Qingdao 266109, China; 2 College of Animal Science and Technology, Qingdao Agricultural University, Qingdao 266109, China; 3 College of Life Science, Qingdao Agricultural University, Qingdao 266109, China; 4 EMF Nutrition Ltd, 715 Marion Street, Winnipeg, MB R2J 0K6, Canada; Institute of Zoology, Chinese Academy of Sciences, CHINA

## Abstract

Oxidative stress (OS), as a signal of aberrant intracellular mechanisms, plays key roles in maintaining homeostasis for organisms. The occurrence of OS due to the disorder of normal cellular redox balance indicates the overproduction of reactive oxygen species (ROS) and/or deficiency of antioxidants. Once the balance is broken down, repression of oxidative stress is one of the most effective ways to alleviate it. Ongoing studies provide remarkable evidence that oxidative stress is involved in reproductive toxicity induced by various stimuli, such as environmental toxicants and food toxicity. Zearalenone (ZEA), as a toxic compound existing in contaminated food products, is found to induce mycotoxicosis that has a significant impact on the reproduction of domestic animals, especially pigs. However, there is no information about how ROS and oxidative stress is involved in the influence of ZEA on porcine granulosa cells, or whether the stress can be rescued by curcumin. In this study, ZEA-induced effect on porcine granulosa cells was investigated at low concentrations (15 μM, 30 μM and 60 μM). *In vitro* ROS levels, the mRNA level and activity of superoxide dismutase, glutathione peroxidase and catalase were obtained. The results showed that in comparison with negative control, ZEA increased oxidative stress with higher ROS levels, reduced the expression and activity of antioxidative enzymes, increased the intensity of fluorogenic probes 2’, 7’-Dichlorodihydrofluorescin diacetate and dihydroethidium in flow cytometry assay and fluorescence microscopy. Meanwhile, the activity of glutathione (GSH) did not change obviously following 60 μM ZEA treatment. Furthermore, the underlying protective mechanisms of curcumin on the ZEA-treated porcine granulosa cells were investigated. The data revealed that curcumin pre-treatment significantly suppressed ZEA-induced oxidative stress. Collectively, porcine granulosa cells were sensitive to ZEA, which may induce oxidative stress. The findings from this study clearly demonstrate that curcumin is effective to reduce the dysregulation of cellular redox balance on porcine granulosa cells *in vitro* and should be further investigated for its protective role against ZEA in animals.

## Introduction

Zearalenone (ZEA) is a toxic compound produced by several species of *Fusarium* and is found to cause mycotoxicosis in animals and humans [1]. It usually exists in contaminated livestock feed consumed by domestic animals, and is subsequently hazardous to the metabolism of organisms remarkably [2, 3]. It is well known that both in females and males, the regulation of estrogen signaling is important in maintaining the normal functionality of reproductive systems [4–6]. Due to their characteristics as highly active nonsteroidal estrogens with hormonal activity, ZEA and its reduced metabolites (such as β-zearalenol) can evidently disrupt the endocrine balance in either females or in males [7–9]. In livestock production, especially in pig production [2–4], both low and high concentrations of ZEA are shown to have adverse effects, leading to hyperestrogenic syndrome, abortion and reproductive failure [10–13]. By liquid chromatography tandem mass spectrometry, ZEA and its metabolites have been detected in naturally contaminated porcine follicular fluid. The concentrations of ZEA and α-zearalenol were about 38.9 pg/mL and 17.6 pg/mL respectively [14]. After a single oral dose of 10 mg/kg b.w. administration, the uptake of ZEA in a pig was estimated to be 80–85% [15]. The dose of 200 mg/kg b.w. ZEA per day, the lowest effective toxic level, affect the development of large follicles via the activation of an apoptotic-like process in the granulosa layer of single mature follicle *in vivo* [12, 16]. In some region, the high concentration contaminations of ZEA had been up to 8,000–10,000 mg/kg in wheat and rice and the notably serious risk of easily contaminated livestock feed should be taken into consideration [17, 18].

Granulosa cells, the somatic cells of mammalian ovarian follicles, have two major functional attributes: steroidogenesis and ovulation [19–21]. Therefore, the failure of oogenesis in the porcine granulosa cells may be one of the most hazardous factors affecting normal porcine reproduction. Recently, Zhu et al. have revealed that relatively high concentration (> 60 μM) of ZEA can induce apoptosis and necrosis in porcine granulosa cells as a result of disrupted steroidogenesis [22].

Oxidative stress is involved in reproductive toxicity caused by various stimuli such as food toxicity [23]. ZEA is one of the toxic compounds existing in contaminated food and has been shown to cause mycotoxicosis. A lots of studies suggest that ZEA can induce oxidative stress in animal cells or tissues [24–27]. However, the precise mechanism underlying the oxidative stress induced by ZEA is poorly understood. In normal conditions, cells generate oxygen species in a balanced way. Once the normal cellular redox balance is broken down, the cells may try to survive by degrading their own protein aggregates or organelles or initiating the process of apoptosis [28]. In process of autophagy or apoptosis, the increase or accumulation of ROS compromises the cellular redox balance. Moreover, antioxidants are inactivated and inhibited to scavenge the free radicals produced in this process. Repression of oxidative stress is believed to be one of the most effective ways in alleviating reproductive disruption caused by ZEA-induced oxidative damage. Therefore, compounds with anti-oxidative activity are usually tested to rescue the disorder of cellular redox balance in organisms.

Curcumin is isolated from the rhizomes of plant *Curcuma longa* and has been used as food additive and traditional medicine in Asia for a long time [29, 30]. It has been shown to be a powerful inhibitor of oxidative stress [30–32]. Many researches have proved that curcumin could directly scavenge free radicals (ROS and RNS) and remove superoxide and peroxide [33, 34]. Recent studies demonstrate that it can alleviate testicular damage by decreasing oxidative stress [35, 36].

The present study was conducted to verify if ZEA can cause oxidative stress at low concentrations in porcine granulosa cells and investigate the effect of curcumin on ZEA-induced oxidative stress. Meanwhile, the study was designated to analyze the gene expression and activities of key antioxidative enzymes in parallel.

## Materials and Methods

### Reagents

ZEA and curcumin were purchased from Sigma (St. Louis, MO). ZEA (Z2125-10MG), a fungal mycotoxin produced by *Fusarium*, binds the estrogen receptor (ER) and its dissolution is used with methanol. Curcumin (C7727-500MG), dissolved by 95% ethanol, a natural phenolic compound, have anti-inflammatory and anti-oxidant properties. Dimethyl sulfoxide (DMSO), M-199 medium, penicillin, streptomycin and fetal bovine serum (FBS) were obtained from Gibco (Carlsbad, CA). Stock solutions of ZEA were prepared by dissolving ZEA in DMSO.

### Animals

The ovaries of mature sows were collected from Qingdao Linhaichun Pig Production Cooperation (Qingdao, Shandong, China). Porcine ovaries of mature pigs were obtained from a local slaughterhouse and maintained at 30–37°C for isolation of granulosa cells. Animals used for ovary collection were euthanized using electrodes. The procedures of animal handling were reviewed and approved by the Ethical Committee of Qingdao Agricultural University (agreement No. 2013–16).

### Isolation and culture of porcine granulosa cells

Granulosa cells were aspirated aseptically from antral follicles (about 4 mm diameter) using a 20 ml syringe (18-gauge needles)[22]. After standing for 10 min, the granulosa cells were centrifuged at 300 g for 5 min according to the methods previously described [22]. The blood cells in the supernatant were aspirated after rinsing with phosphate-buffered saline (PBS). Then the granulosa cells were cultured in M-199 medium supplemented with 10% FBS and 1% penicillin-streptomycin in a humidified incubator with 5% CO_2_ at 37°C [37, 38].

### Drug treatments

Granulosa cells were seeded into wells in 6-well plates at a density of 2 ╳ 10^4^ cells per well. To study the generation of oxidative stress in the ZEA-treated granulosa cells, ZEA was added to the medium at final concentrations of 15, 30 and 60 μM and the cells were incubated for 8, 16 and 24 h. To investigate the protective mechanism of curcumin against ZEA-induced intracellular ROS, cells were pre-treated with or without 20 μM curcumin for 12 h and then co-treated with 60 μM ZEA for 24 h. Control groups set up with DMSO at the same concentrations treated with ZEA or co-treated with curcumin is to guarantee the accuracy of experimental design.

### RNA purification and quantitative real-time PCR

Porcine granulosa cells were harvested and extracted for total RNA using a RNA extraction kit (TaKaRa, Dalian, China) according to the manufacturer’s protocols. After reverse transcription to cDNA (TransScript One-Step, Beijing, China), quantitative real-time PCR (qRT-PCR) reaction was carried out on a Light Cycler real-time PCR instrument (Roche LC480, Germany) using a Light Cycler VR SYBR Green I Master (Roche, 04887352001, Germany). Primers used are listed in Table 1. Amplification was performed in 10 μL reactions containing 1 μL of cDNA, 5 μL of SYBR green master mix, 0.4 μL each of primers (10 μM), and 3.6 μL of nuclease-free water as according to the manufacturer’s recommendations. PCR reactions were initialed at 95°C for 10 min, followed by 55 cycles of denaturing at 95°C for 10 s, annealing at 60°C for 30 s, and finally a cooling step at 4°C. The gene expression was normalized to β-actin as reference using the formula: 2^^-(target gene CT value—reference gene CT value)^. Relative fold changes were calculated in comparison with the level in DMSO group (control group), and amplifications were performed in triplicate with mRNA from at least three independent experiments.

**Table 1:** Sequences of primer sets used for gene expression analysis.

Gene name	Abbreviation	Forward 5’-3’	Reverse 5’-3’
Superoxide dismutase 1	Sod1	AAGATTCTGTGATCGCCCTCT	ACTTCCAGCATTTCCCGTC
Glutathione peroxidase	Gpx	AGTCGGTGTATGCCTTCTCG	AGCTCGTTCATCTGGGTGTAGT
Catalase	Cat	TTAGTGCTCCCGAACAGACG	CACTGAAGTTCTTGACCGCTTT
Actin, beta	β-Actin	CCACGAAACTACCTTCAACTCC	CCTGCTTGCTGATCCACATC

### Flow cytometric analysis of intracellular reactive oxygen species (ROS)

Cell-permeable fluorogenic probe 2’, 7’-Dichlorodihydrofluorescin diacetate (DCFH-DA) (Beyotime, Nantong, China), could freely enter into cell. However, it produces highly fluorescent 2’, 7’- dichlorodihydrofluorescein (DCF) in cells, after interacting with intracellular nonspecific esterase. It has been widely used as indicator of intracellular reactive oxygen species (ROS) via fluorescence staining.

After 24 h incubation, the cells were trypsinized and collected, then rinsed 3 times using PBS, incubated with 10 μM DCFH-DA at 37°C for 20 min according to the manufacturer’s instructions. DCF fluorescence was detected by flow cytometry (Becton Dickinson) at excitation wavelength of 488 nm and emission wavelength of 525 nm. For each sample, 20,000 events were recorded.

Dihydroethidium (DHE) fluorescent probe (Beyotime) was used for detecting superoxide anion (O_2_^-^) in the granulose cells, which were dehydrated and showed red signals. Harvested cells were incubated with 10 μM DHE for 30 min at 37°C according to the manufacturer’s instructions. Measurement of red fluorescence was carried out on flow cytometry at excitation wavelength of 535 nm and emission wavelength of 610 nm. Flow cytometric was conducted in duplicate in at least three independent experiments.

### Light and fluorescence microscopy imaging of porcine granulosa cells

To evaluate the growth of porcine granulosa cells, light microscope was employed. Because the cells were seeded into 6-well plate, data were collected directly over time. 24 h after treatment, ROS in the granulosa cells was measured by incubating the cells with the probes DCFH-DA and DHE. The intensity of fluorescence was recorded by a fluorescence microscope.

The granulosa cells were randomly selected in five different areas of each dish and the dead or floated cells were calculated under microscope. The calculations were conducted in duplicate in at least three independent experiments.

### Enzyme activity

Porcine granulosa cells incubated for 24 h in media containing different concentrations of ZEA were collected and assayed for the activities of superoxide dismutase1 (SOD1), catalase (CAT), glutathione peroxidase1 (GPX1) and glutathione (GSH), using kits from Nanjing Jiancheng Bioengineering Institute (Nanjing, China) according to the manufacturer’s protocols. Protein concentration was measured using Eppendorf Biophotometer Plus (Eppendorf.AG). After mixing the intracellular homogenate with reagents, the samples were incubated at 37°C overnight for multiscan spectrum (Bio-Rad) testing. All samples were run in duplicate in at least three independent experiments.

### Statistical analysis

All data are presented as mean ± SD. Comparisons among groups were performed by the Student’s *t*-test or one-way analysis of variance (ANOVA) followed by the Tukey test, while for multiple comparisons, Graph-Pad Prism analysis software (Graph-Pad Software, San Diego, CA) was applied. Difference was considered significant if *P* < 0.05 or highly significant if *P* < 0.01.

## Results

### Effect of ZEA on the growth of porcine granulosa cells

**Fig 1:**
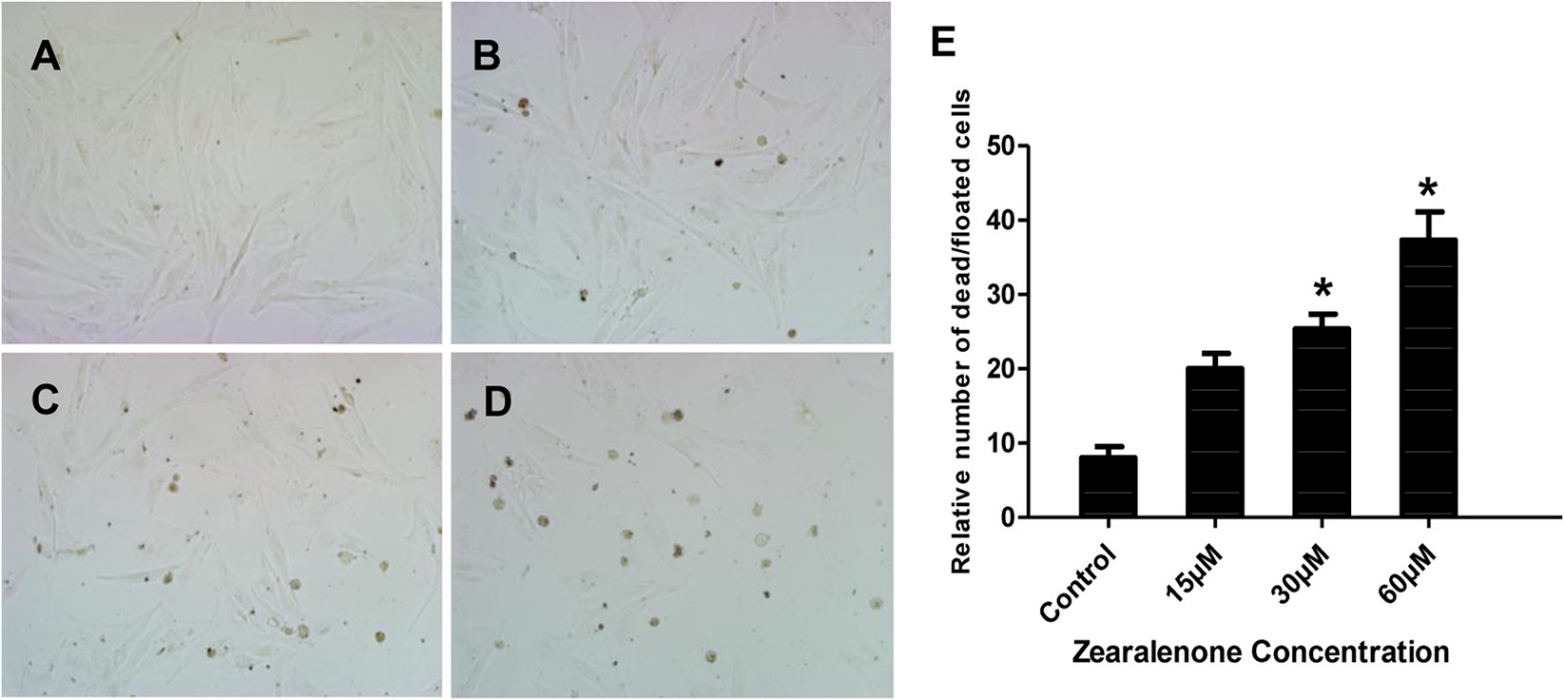
Effect of zearalenone on the growth of porcine granulosa cells. The Porcine granulosa cells were treated with ZEA (15, 30 and 60 μM) for 24 h and imagined by microscope on 6-well plates. A. Untreated cells (Control). B. 15 μM ZEA-treated cells. C. 30 μM ZEA-treated cells. D. 60 μM ZEA-treated cells. E. Quantification of dead and floated cells after treatment of various ZEA concentrations for 24 h. Asterisk (*) indicates significant difference (P < 0.05).

To investigate whether low concentrations of ZEA affect the growth of porcine granulosa cells, microscope was used to record the cells following different periods of ZEA treatment (8–24 h). Cells were isolated and cultured in 10 mL media in a 10-cm dish. After culture, the cells were seeded into wells of 6-well plates at a density of 2 × 10^4^ cells per well. After cultured for 24 h, the cells were incubated with ZEA ranging from 15 μM to 60 μM for 24 h. The growth conditions of treated cells are shown in Fig 1. Adherent cells were in poor condition and had more dead and floated cells as ZEA concentrations increased within the 24 h observation period. When incubated for 16 h, no dead cells were observed (data not shown), suggested that ZEA is noxious to the cells.

### Gene expression of antioxidative enzymes in porcine granulosa cells exposed to ZEA

**Fig 2:**
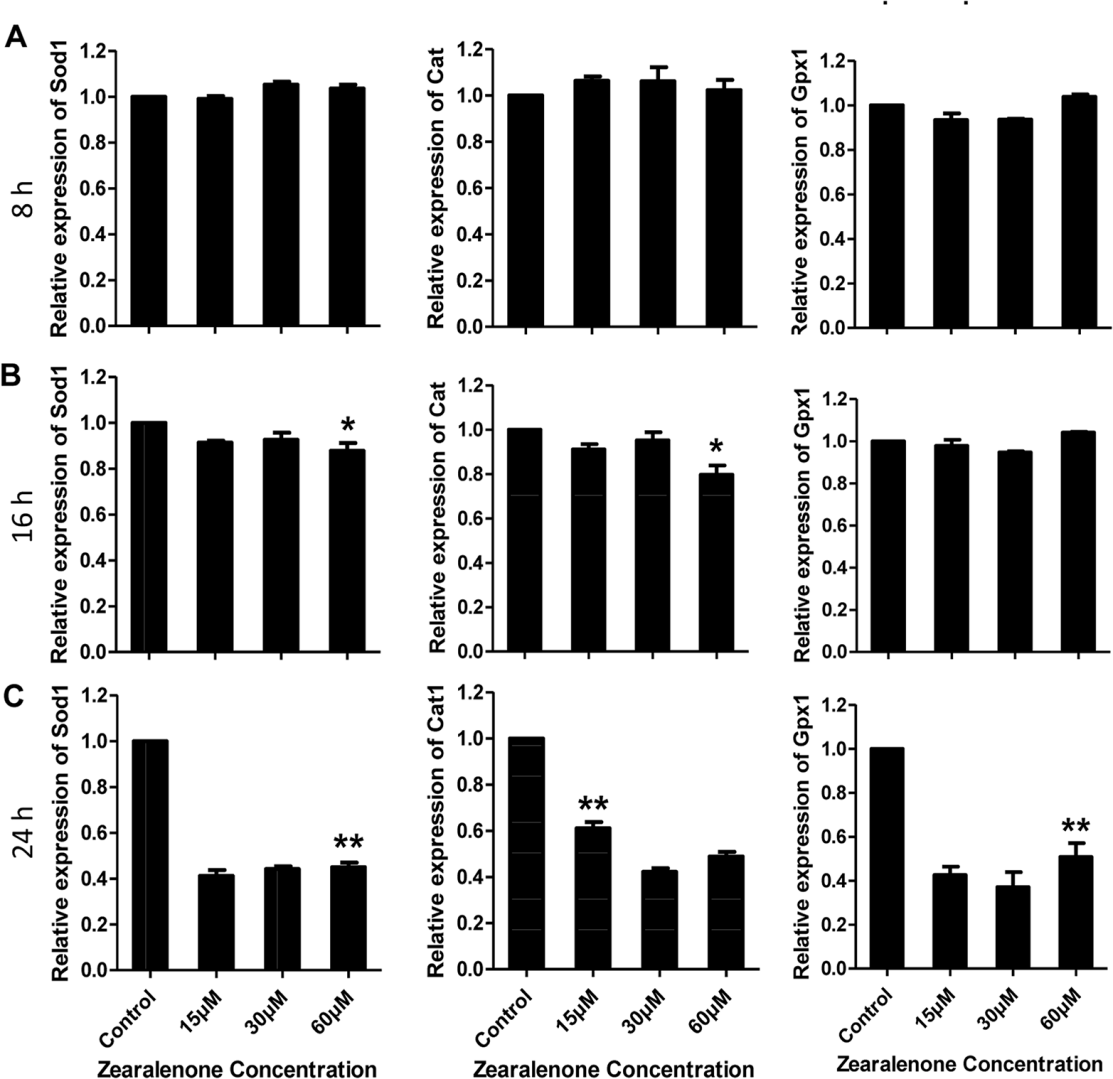
The expression levels of endogenous antioxidative enzymes Sod1, Cat and Gpx1 in porcine granulosa cells treated with ZEA for different times (8–24 h). A-C. Gene expression in ZEA-treated cells at 8 h, 16 h and 24 h. The expression level was normalized to that of β-actin as internal control. Compared to the control group, relative fold changes were presented as mean ± SD. All experiments were repeated at least three times independently. Asterisk (*) indicates significant difference (P < 0.05), while asterisk (**) represents highly significant difference (P < 0.01).

Gene expression of antioxidative enzymes in the porcine granulosa cells were determined after exposure to different ZEA concentrations for different times, to examine if ZEA would alter the oxidic status in the granulosa cells. QPCR was conducted to quantify the expression of antioxidative enzymes, Sod1, Cat and Gpx1 in DMSO control and ZEA-treated cells. After ZEA treatment for 8 h, no apparent change in the expression of these genes was observed in treated granulosa cells as compared with the control (Fig 2A). At 16 h, the expression of Sod1 and Cat decreased significantly only in the cells treated with 60 μM ZEA in comparison to negative control, whilst the expression of Gpx1 was not affected at any ZEA concentration used (Fig 2B).

With the above findings, we investigated whether the lower concentrations of ZEA would affect the expression of antioxidative enzymes in longer treatment time. The results (Fig 2C) showed significant changes in the expression of these genes, even Gpx1, at various ZEA concentrations and durations of treatment.

### ROS levels in porcine granulosa cells exposed to ZEA

Based on the expression of antioxidative enzymes, we assumed that ZEA might affect the intracellular ROS levels in the porcine granulosa cells. ROS was then assayed using fluorescent probes DCFH-DA and DHE.

**Fig 3:**
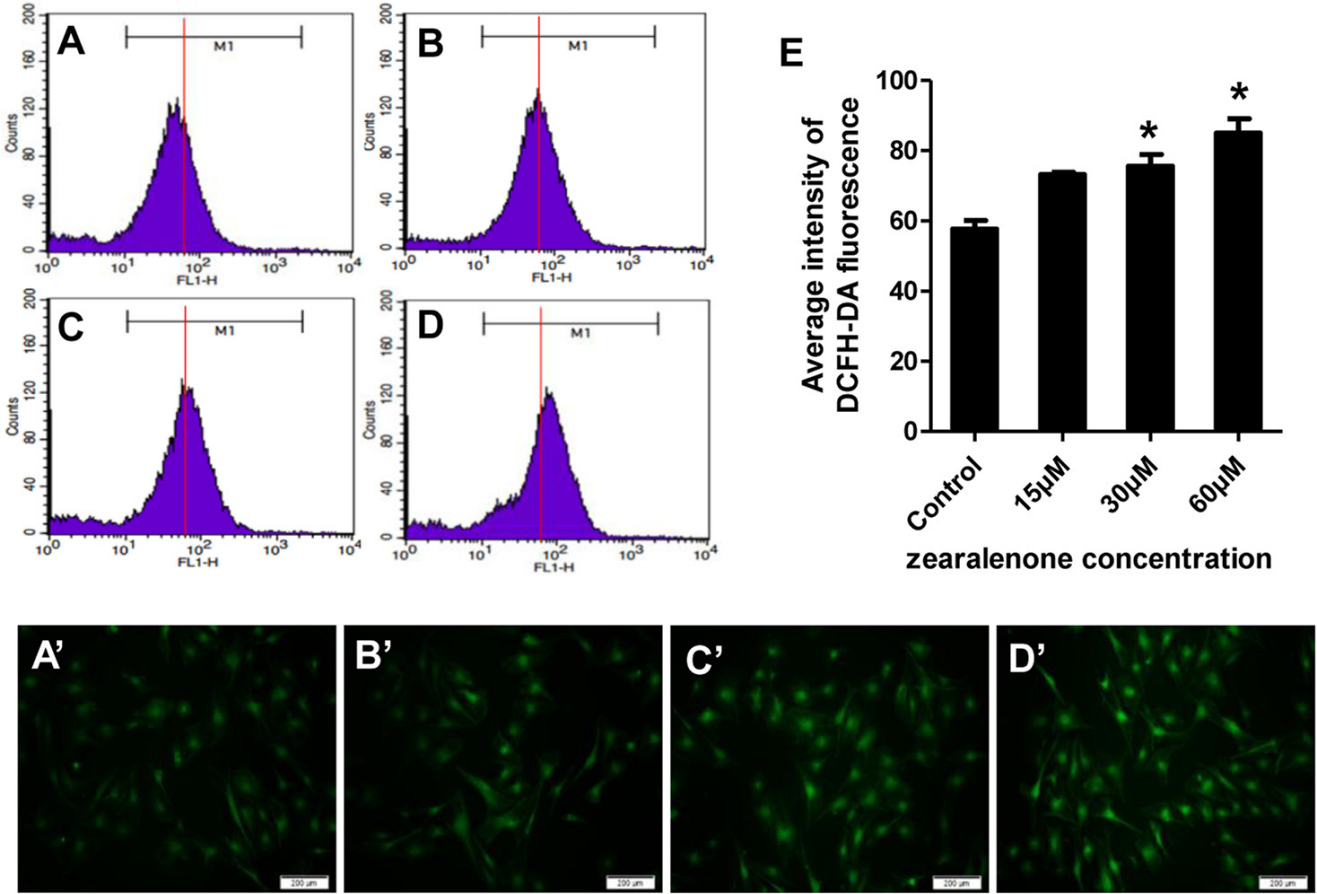
Intracellular ROS levels assayed with DCFH-DA fluorescent probe in porcine granulosa cells treated with ZEA. A (A’), Control (untreated); B (B’), 15 μM ZEA; C (C’), 30 μM ZEA; D (D’), 60 μM ZEA. Intracellular ROS was measured by flow cytometry (A-D) and fluorescent imaging (A’-D’) using DCFH-DA probe. For better clarity, a guidance line (red) is drawn through histograms for comparison. E, Average intensity from DCFH-DA. The results are presented as mean ± SD. Asterisk (*) indicates significant difference (P<0.05).

DCFH-DA fluorescent probe could freely cross cell membrane, which is oxidized to a fluorescent DCF by intracellular ROS. The levels of intracellular ROS could be quantified by the intensity of DCFH-DA fluorescence (Fig 3A). Compared with the control, the fluorescent intensity was enhanced by comparing with the guidance line (red) as the standard, indicating that the intracellular ROS level was significantly increased. When the granulosa cells were treated with ZEA for 24 h and then incubated for 20 min on 6-well plates, with decreased ZEA concentrations, fluorescent intensity increased in adherent granulosa cells loaded with DCFH-DA probe (Fig 3A’–3D’). The results demonstrated that the intercellular ROS in the cultured porcine granulosa cells could be up-regulated by ZEA.

**Fig 4:**
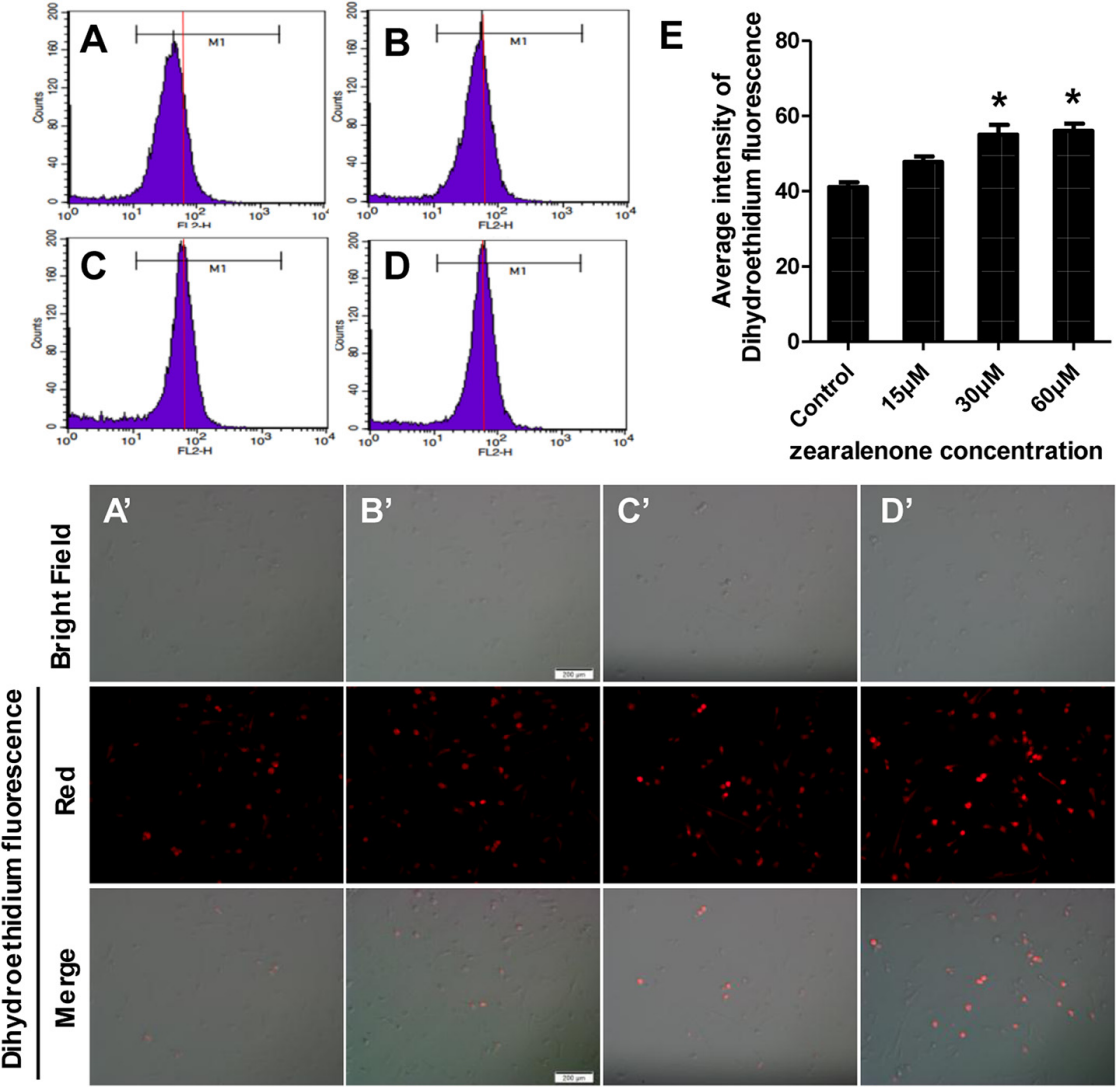
Intracellular ROS levels assayed with dihydroethidium fluorescent probe in porcine granulosa cells treated with zearalenone. A (A’), Control (untreated); B (B’), 15 μM ZEA; C (C’), 30 μM ZEA; D (D’), 60 μM ZEA. Intracellular ROS was measured by flow cytometry (A-D) and microscope and fluorescence imaging (A’-D’) by detecting the signals of superoxide anion in cells. For better clarity, a guidance line (red) is drawn through histograms for comparison. E, Average intensity of dihydroethidium. The results are expressed as mean ± SD. Asterisk (*) indicates significant difference (P < 0.05)

DHE is one of the most common used fluorescence probes for superoxide anion. Once dehydrogenation occurs, the red fluorescence from the probe can be detected as the signals of superoxide anion, announcing the levels of intracellular ROS. The data from flow cytometry shown in Fig 4A–4D indicated that ROS in the granulosa cells increased based on the intensity of red fluorescence emitted from DHE. Similar effects of ZEA on the porcine granulosa cells could be distinguished from microscope and fluorescence images in Fig 4A’–4D’. Collectively, ZEA treatments resulted in a significant increase of intracellular ROS in porcine granulosa cells.

### Activities of antioxidative enzymes in porcine granulosa cells exposed to ZEA

**Fig 5:**
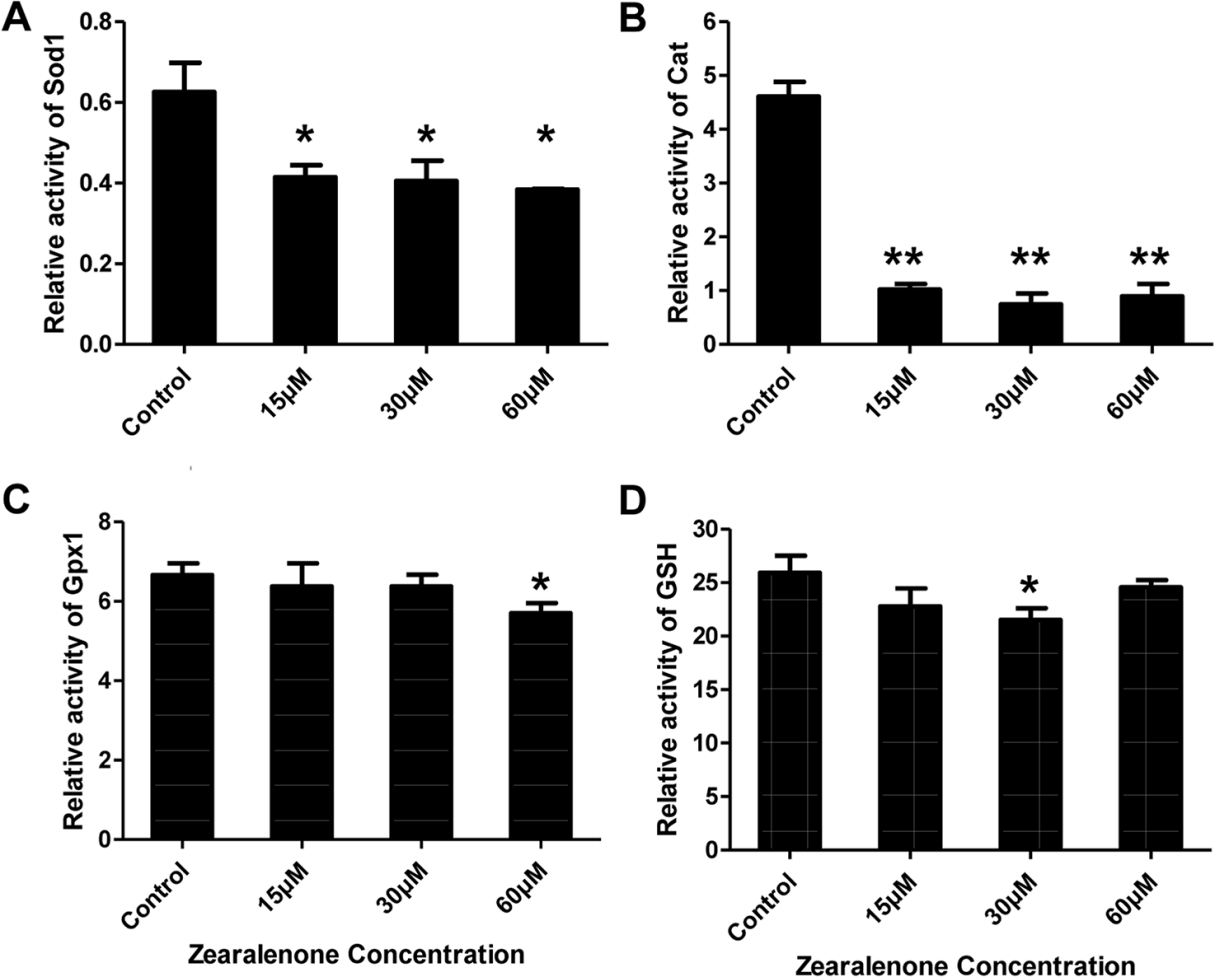
The enzymatic activities of SOD1, CAT, GPX1 and GSH in porcine granulosa cells. After 24 h of ZEA treatment, porcine granulosa cells were collected for measurement. All values were normalized to protein level in a loading control and presented as relative fold changes in comparison to untreated control. All experiments were repeated at least three times. Asterisk (*) indicates significant difference (P < 0.05), asterisk (**) represents highly significant difference (P < 0.01).

It is essential to determine whether ZEA affects the activities of SOD1, CAT, GPX1 and GSH, compared to the control. As shown in Fig 5A, the activity of SOD1 remarkably decreased in the granulosa cells, indicating that the activity of intracellular SOD1 in the granulosa cells was significantly inactivated by ZEA (Fig 5A) and the disputation of superoxide failed to eliminate the underlying oxidative stress. Furthermore, the activity of CAT in the cells was highly and significantly lower in comparison to the untreated cells (Fig 5B). As for the activity of GPX1, only 60 μM ZEA showed significant inhibition in the treated cells (Fig 5C). When the concentration of ZEA was reduced to 30 μM, GSH was significantly higher than at the other levels (Fig 5D). Briefly, in contrast with SOD1 and CAT, the activities of GPX1 and GSH were not significantly different among all groups, but the activities of antioxidative enzymes were down-regulated with increasing ZEA concentration.

### Effect of curcumin on ZEA-induced oxidative stress in porcine granulosa cells

**Fig 6:**
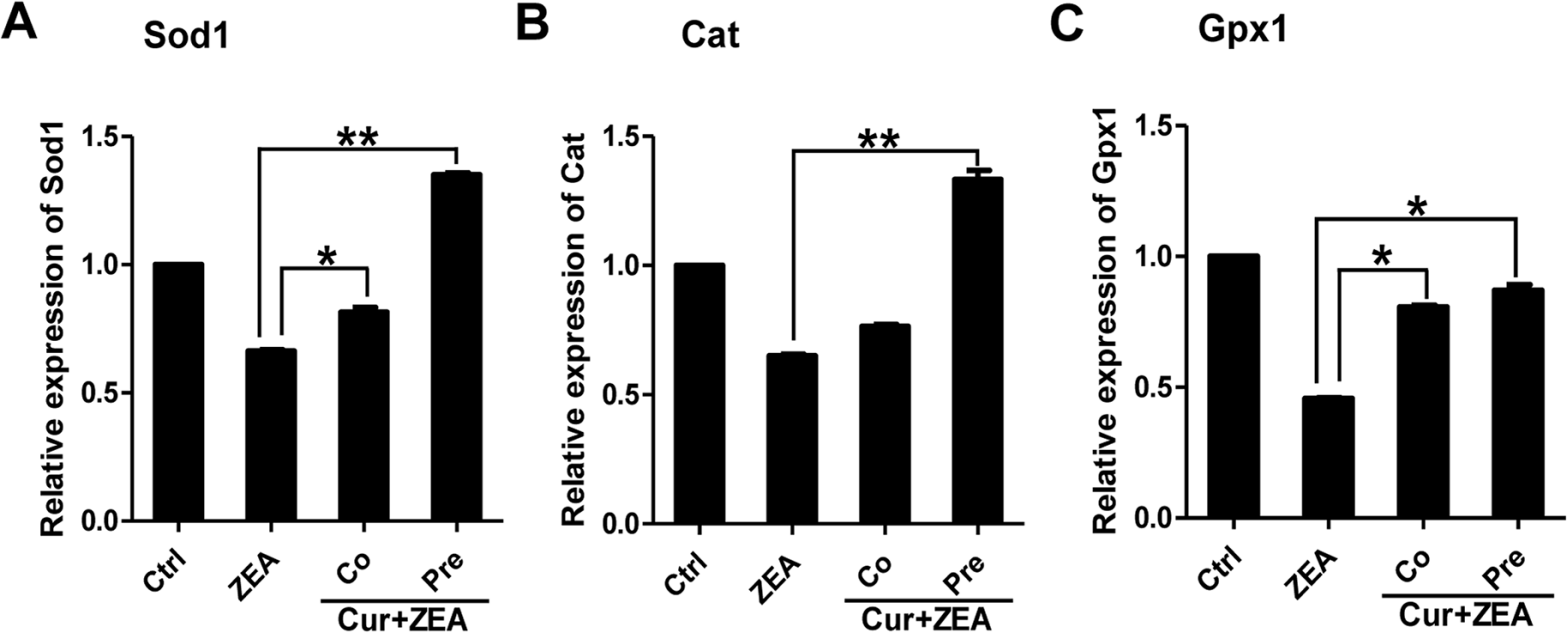
The expression of endogenous antioxidative enzymes Sod1, Cat and Gpx1 in porcine granulosa cells treated with DMSO (control group); ZEA (60 μM ZEA treatment for 24 h); Cur+ZEA (Co, co-treatment with curcumin and ZEA; Pre, pre-treatment with curcumin for 12 h). Gene expression was normalized to β-actin as a loading control. Compared to the control group, relative fold changes are presented as mean ± SD. All experiments were repeated at least three times. Asterisk (*) indicates significant difference (P < 0.05) while asterisk (**) indicates highly significant difference (P < 0.01).

Curcumin has potent anti-oxidation ability and can be used to rescue oxidative stress in cells. After determination of the non-cytotoxic response, low (20 μM) and high (200 μM) concentrations of curcumin were used. Measurements showed that at the low concentration, curcumin exhibited antioxidative effects, but at the high concentration, it increased oxidative stress (data not shown). To investigate the protective mechanism of curcumin on ZEA-induced oxidative stress in porcine granulosa cells, different treatments were tried, such as pre- and co-treatments of curcumin with ZEA (Cur+ZEA). QPCR was employed to quantify the expression of antioxidative enzymes SOD1, CAT and GPX1 in these treated granulosa cells. As shown in Fig 6, the expression of SOD1 and CAT was significantly increased in the cells pre-treated with curcumin compared to the cells treated with ZEA only. However, Co-treatment of curcumin and ZEA did not show significantly protection in maintaining normal cellular redox balance in the ZEA-treated cells; pre-treatment of curcumin exhibited antioxidative function against oxidative stress, although the expression of GPX1 was not significantly different in comparison to ZEA group. These results demonstrate that curcumin could rescue the oxidative stress to the level observed in control group (Fig 6C).

**Fig 7:**
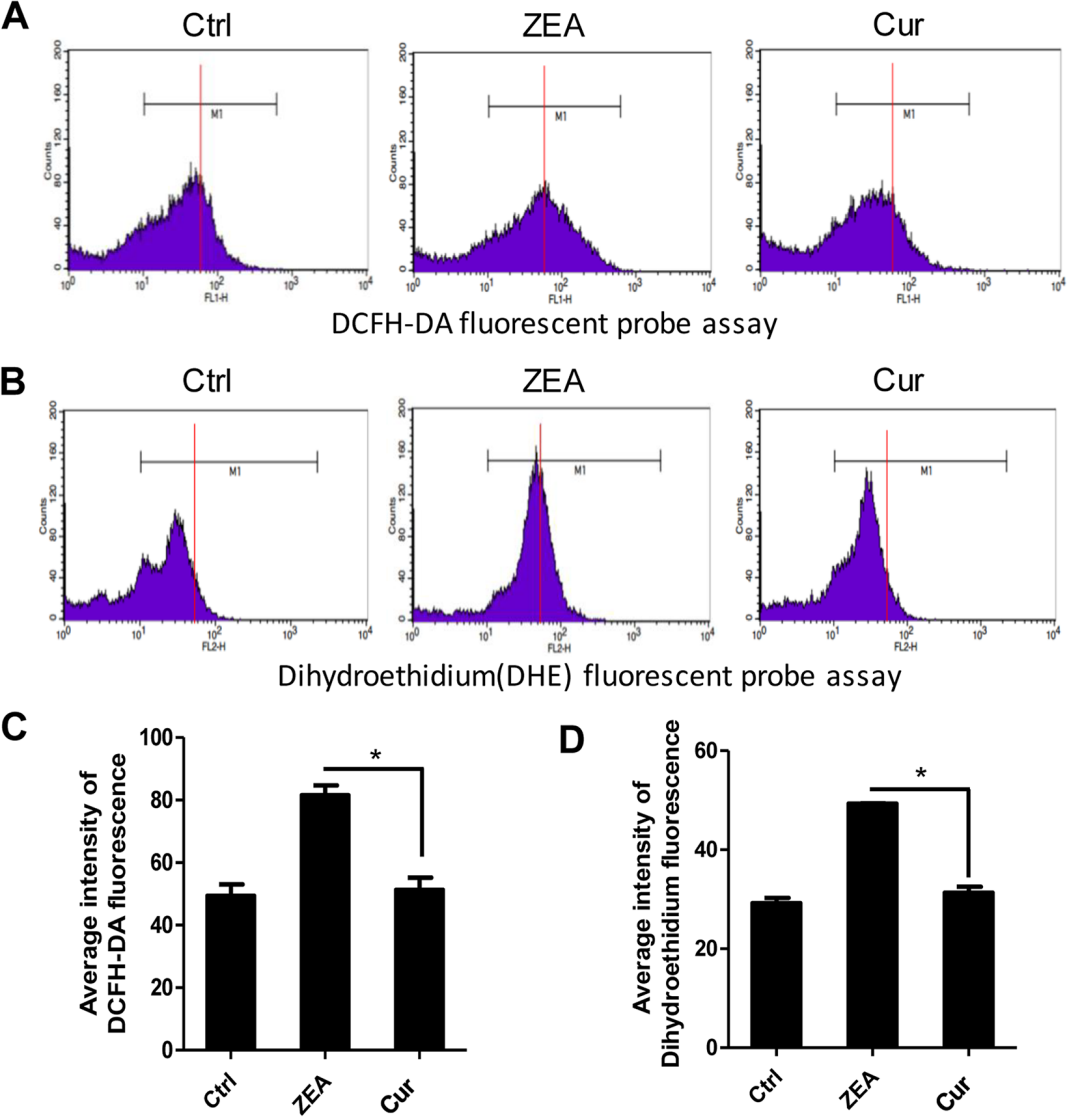
Intracellular ROS levels assayed using DCFH-DA and DHE fluorescent probes in porcine granulosa cells treated with curcumin. A-B, Intracellular ROS was measured by flow cytometry (DCFH-DA and DHE). For better clarity, a guidance line (red) is drawn through histograms for comparison. C-D, Average intensity of DCFH-DA and DHE fluorescence in porcine granulosa cells treated with curcumin. The results are presented as mean ± SD. Asterisk (*) indicates significant difference (P<0.05)

**Fig 8:**
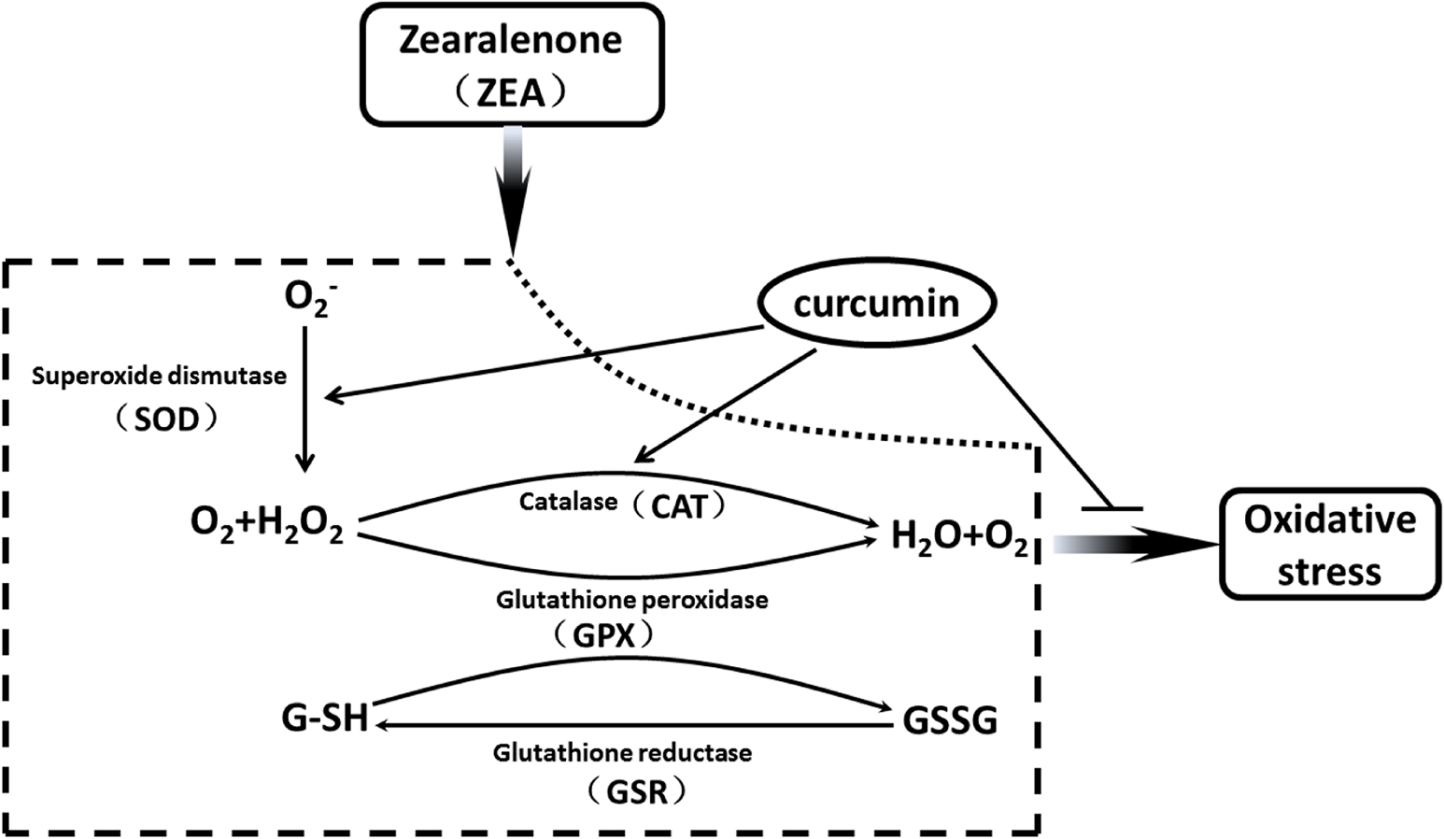
The rescue of curcumin of oxidative stress induced by ZEA in porcine granulosa cells. Multiple enzyme systems are involved in the effect of ZEA, SOD1 in the cytoplasm dismutates O_2_^-^ to H_2_O_2_ and water. GPX1and CAT converts H_2_O_2_ to water. During this process, GSH is oxidized to the disulfide form (GSSG) by GPX1, which is converted back to GSH through glutathione reductase action (GSR). ZEA stimulated these enzyme systems, resulting in oxidatvie stress in porcine granulosa cells. Curcumin rescued cellular redox balance through inhibition of SOD1 and CAT signal pathway in porcine granulosa cells after pre-treatment for 12 h.

After measuring the expression of antioxidative enzymes genes, we examined that the intracellular ROS levels in the porcine granulosa cells treated with ZEA (60 μM) and pretreated with curcumin (20 μM). ROS assays were carried out using both DCFH-DA and DHE. The fluorescent intensity was determined according to the guidance line (red) drawn on the histograms (Fig 7A and 7B). The fluorescent intensity in cells pre-treated with curcumin was weaker than that in the ZEA-treated cells, but similar to that of the control (Fig 8C and 8D).

## Discussion

The toxicity of ZEA has attracted intensive attention due to the oestrogenic effects of its metabolic products. Previous studies show that ZEA exists in the contaminated livestock feed and food, and potentially impacts human and animal health [39–43]. For domestic animals, ZEA seriously interferes with their reproduction [40–42] and induces oxidative stress as a result of decreased antioxidative enzymes [44, 45]. However, no detailed information regarding ROS induced by ZEA in porcine granulosa is available, and the rescue of the oxidative stress has not been attempted.

Curcumin possesses antioxidative properties and are shown to inhibit oxidative stress [33, 34] and generation of ROS *in vivo* [46]. Many studies reveal that curcumin also has protective effect on ionizing radiation-induced ovarian toxicity [47] and testicular damage [36, 48]. However, the ameliorative effect of curcumin largely depends on its concentration. At high concentration, it could lead to cell death [49].

This study finds that ZEA can induce oxidative stress in porcine granulosa cells. Whether curcumin would reduce the generation of ROS induced by ZEA was investigated following the treatment of cells using lower concentrations of ZEA as described in previous study [22]. Gene expression and activities of key antioxidative enzymes increased in the granulosa cells treated with various concentrations of ZEA. ROS is consisted of free radicals such as superoxide anion radical (O_2_^-^) and non-free radical ROS, including hydrogen peroxide (H_2_O_2_) [50]. With the enrichment of O_2_^-^ and H_2_O_2_, oxidative stress occurs, and the cellular redox balance is impaired, resulting in oxidative damage to cells [51, 52]. Multiple enzyme systems are involved in ZEA toxicity, SOD1 in the cytoplasm dismutates O_2_^-^ to H_2_O_2_ and water, GPX1 and CAT then convert H_2_O_2_ to water (Fig 8). This study confirmed that ZEA induces oxidative stress by impairing these two processes. By contrast, curcumin executes good protection to rescue the destroyed pathways (Fig 8). During the process of reducing peroxides, GPX1 also plays a role in oxidizing GSH to a disulfide form (GSSG), which is converted back to GSH by the action of glutathione reductase (GSR) (Fig 8) [23, 53]. Oxidative stress in oocyte inhibits nuclear and cytoplasmic maturation [54] and may result in apoptosis [55, 56]. Study shows that the oxidative stress induced by di (2-ethylhexyl) phthalate (DEHP) inhibits the growth of ovarian antral follicles [57].

Granulosa cells as a primary unit of ovarian follicle are critical to guarantee the maturation of oocyte and maintain the normal level of hormones in animal. Due to the hormone disruption by ZEA, the proliferation of porcine granulosa cells decreases while as the ratio of apoptosis enhances at high concentrations of ZEA [22]. The growth of cells was found worse following the treatment of ZEA at low concentrations. Enzyme activities associated with oxidative stress have been well documented [54, 57, 58]. With the decrease of expression of antioxidative enzymes (SOD1, GPX1, CAT), the intensity of ROS in the granulosa cells emitted from the fluorescence probes (DCFH-DA, DHE) increased. Due to the rescue of oxidative stress by curcumin, the expression of antioxidative enzymes and the levels of ROS were decreased accordingly. It is obvious that the activities of antioxidative enzymes were inactivated in the porcine granulosa cells by ZEA. In summary, our results show that oxidative stress could be induced by ZEA in porcine granulosa cells and could be rescued by curcuma. This would provide insight into the possibility to manipulate reproductive homeostasis via regulating the cellular redox balance in gonadal somatic cells.
